# Supplementary material for: Control of compound leaf patterning by MULTI-PINNATE LEAF1 (MPL1) in chickpea
Source: Nat Commun. 2023 Dec 7;14:8088. doi: 10.1038/s41467-023-43975-9 (PMC10703836; doi:10.1038/s41467-023-43975-9)
Supplement: Supplementary file 1 — Supplementary Information [file 41467_2023_43975_MOESM1_ESM.pdf]

Control of compound leaf patterning by *MULTIPLE-PINNATE LEAF1* (*MPL1*) in chickpea

Liu et al.

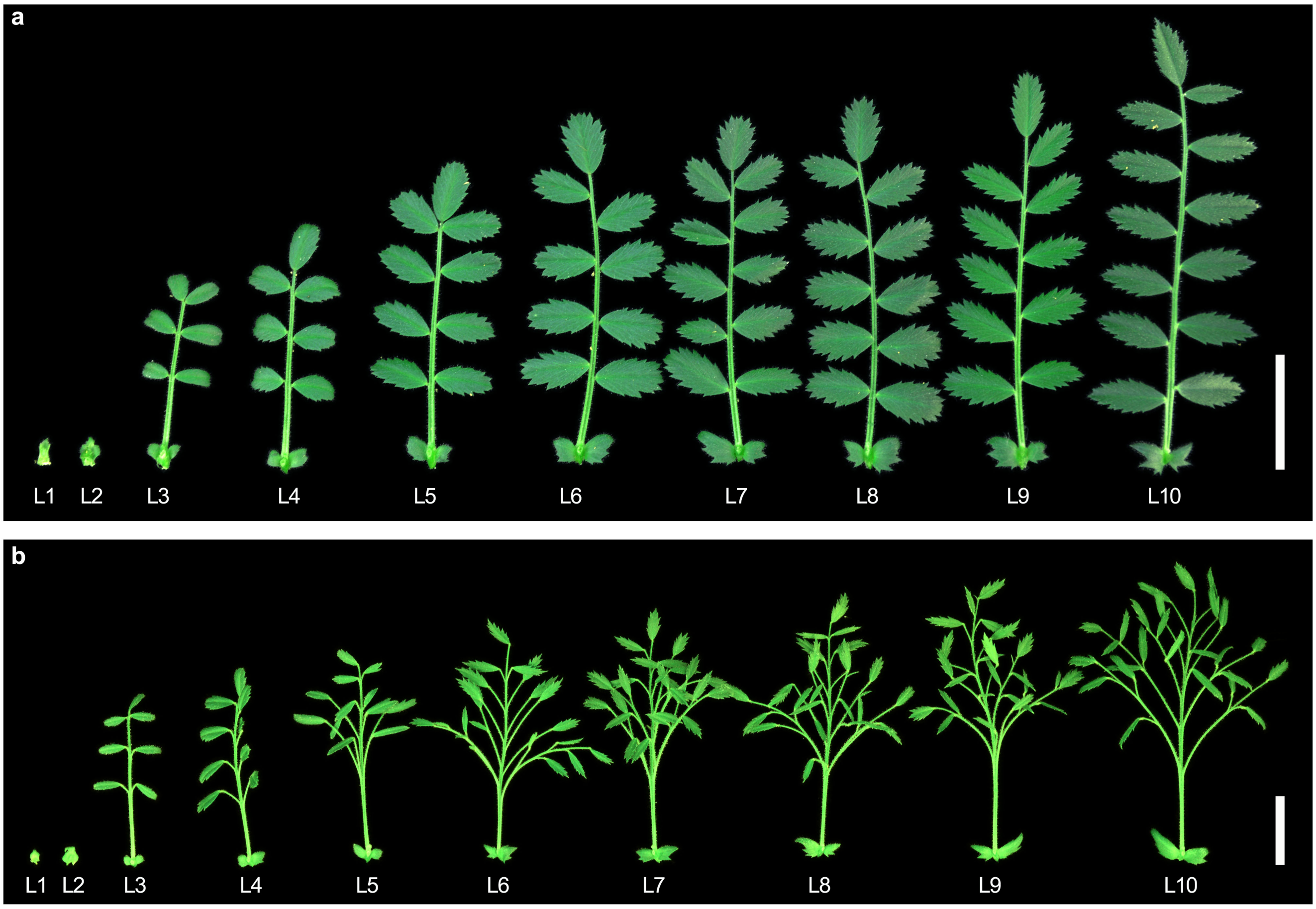

Supplementary Fig. 1. Heteroblastic leaf series of WT and *mpl1-1.* Leaf morphology in successive nodes from 1 to 10 in 6-week-old wild-type (**a**) and *mpl1-1* (**b**) plants. The juvenile leaf with a single leaflet developed on the nodes 1 and 2 in both WT and *mpl1-1*. Each successive leaf became increasingly compound in both WT and *mpl1-1*, with *mpl1-1* exhibiting more complex leaf structures than WT from node 5 onwards. Bars: 2 cm.

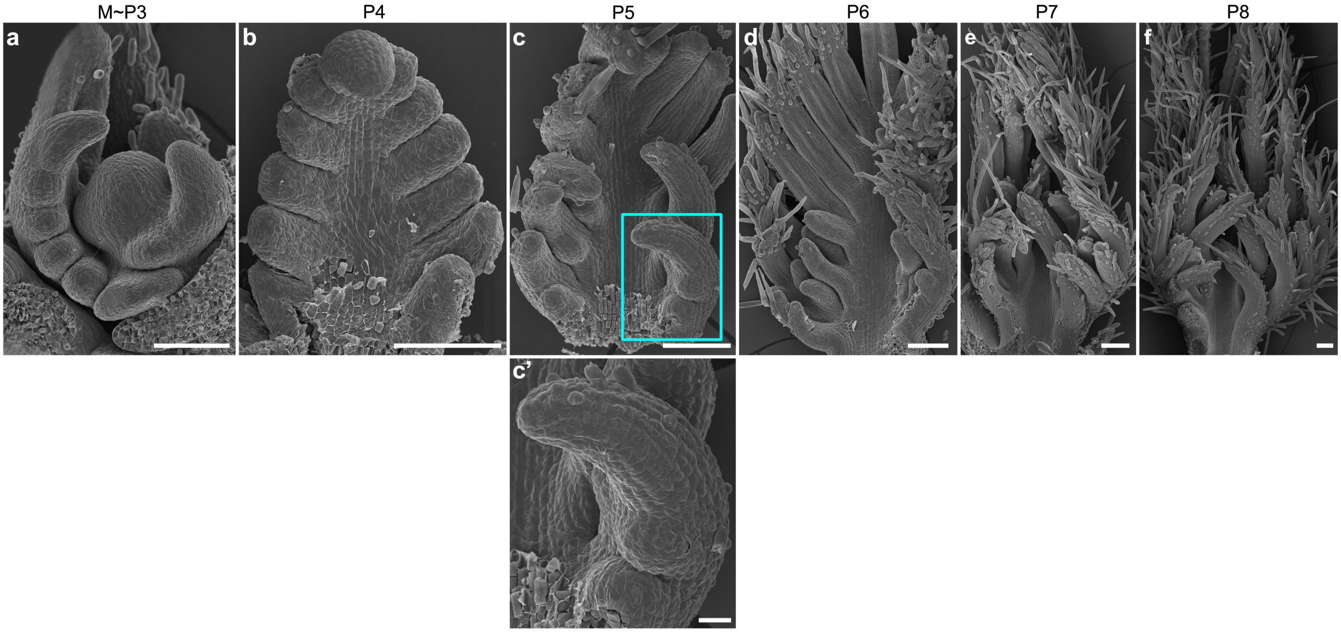

Supplementary Fig. 2. Scanning Electron Microscopy analysis of compound leaf development in *mpl1-1* mutant. a, Shoot apical meristem (SAM) with three visible leaf primordia (P1-P3). **b-f,** subsequent leaf primordia P4-P8 of the *mpl1-1* mutant. **c',** A close-up view of (**c**). Scale bars, 100 µm in **a-f**; 20 µm in **c'**. Similar results were obtained from three biological replicates for each tissue or organ.

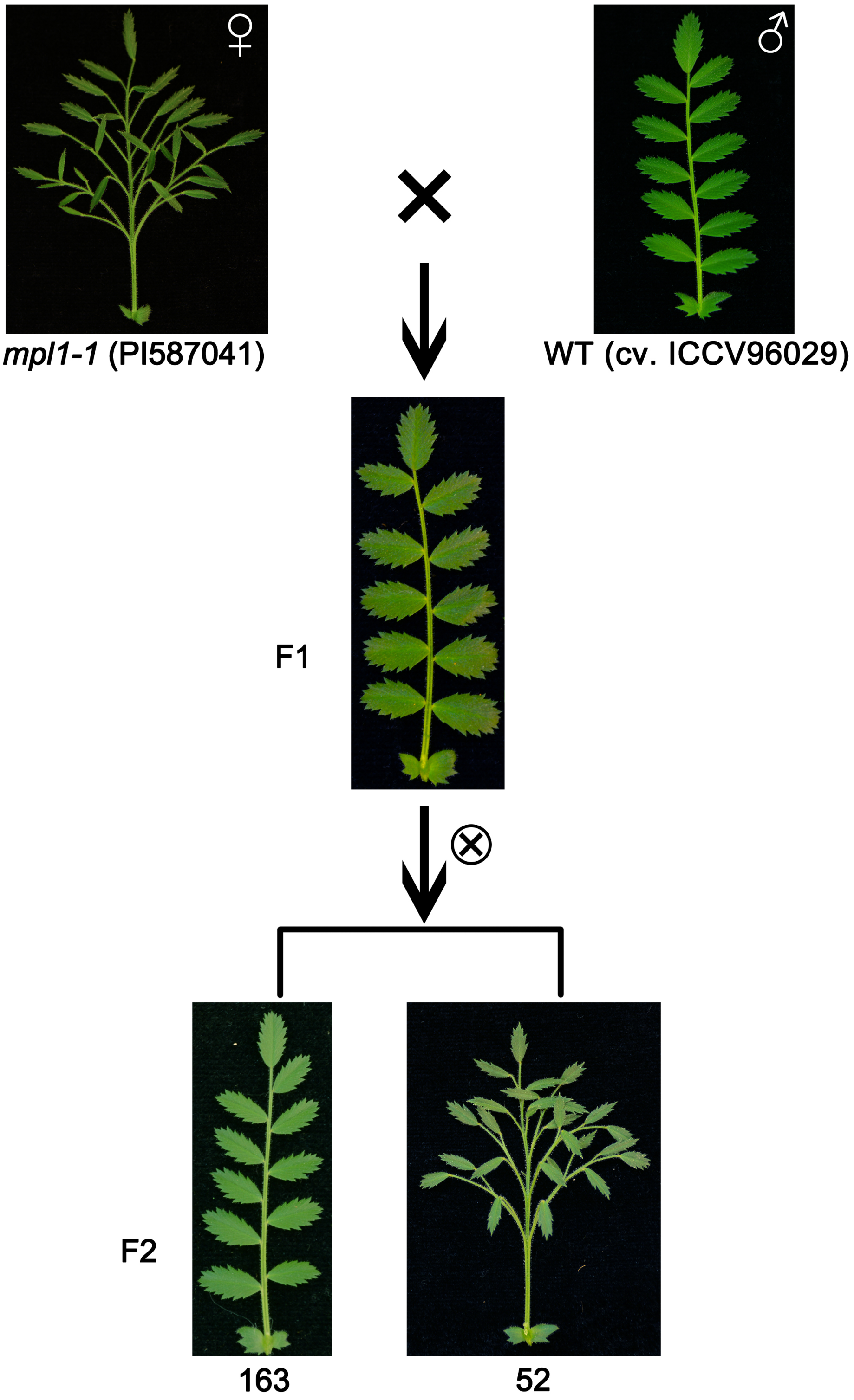

Supplementary Fig. 3. Segregation analysis of *mpl1* mutant and WT in F2 population. F1 plants displayed a WT leaf phenotype, while the F2 population from self-pollination of F1 backcrossed plants showed a 3:1 segregation ratio of WT and *mpl1-1* mutant.

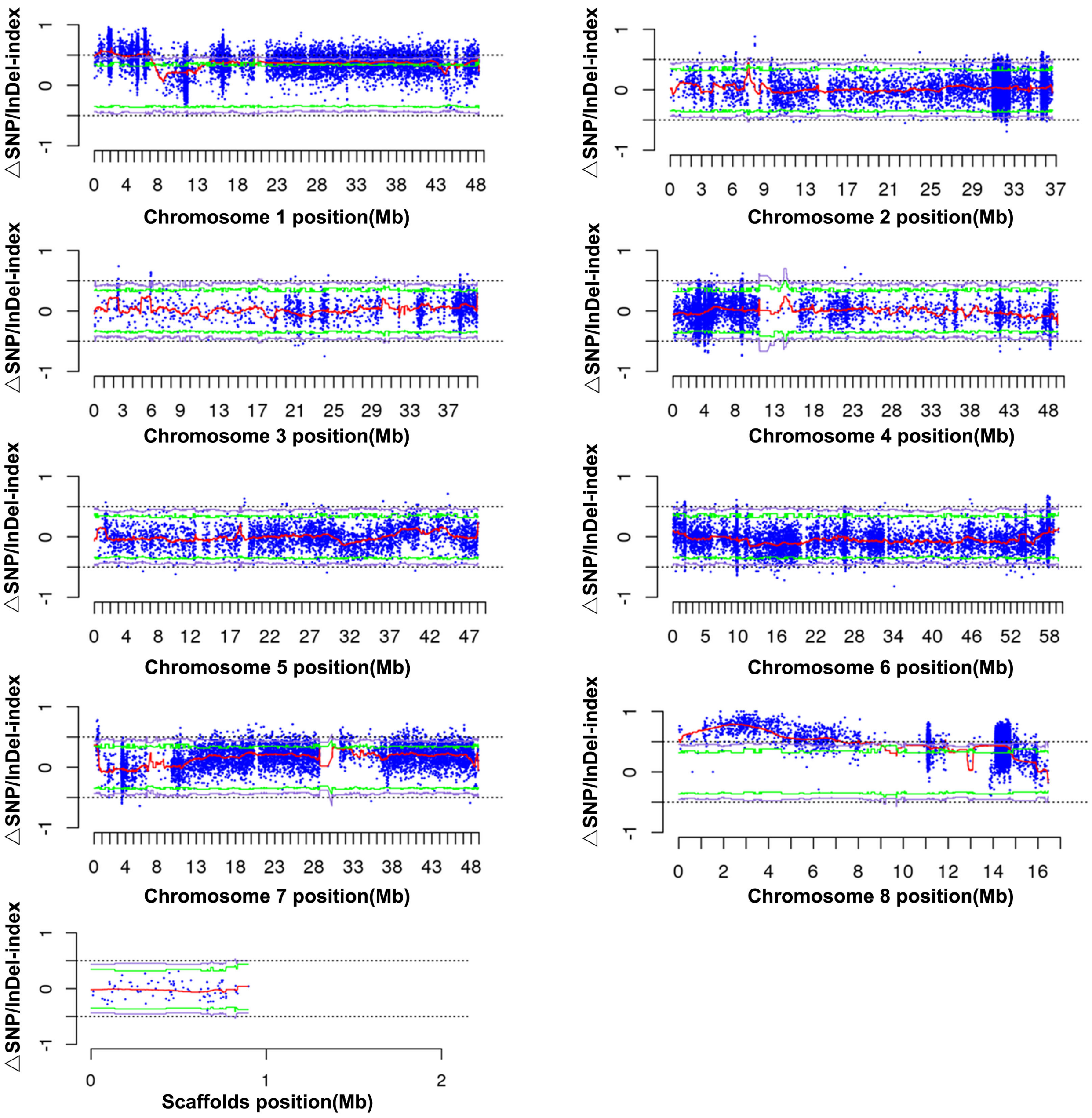

Supplementary Fig. 4. Bulk Segregant Analysis (BSA) of delta SNP/InDel (ΔSNP/InDel)-index between extreme pools grouped by chromosomes. The x-axis corresponds to the chromosomal position, and the y-axis represents the value of the ΔSNP/InDel-index. The red line represents the mean value of the ΔSNP/InDel-index along the chromosomes. The green and gray lines represent the corresponding two-sided 99% and 95% confidence intervals for the ΔSNP/InDel-index.

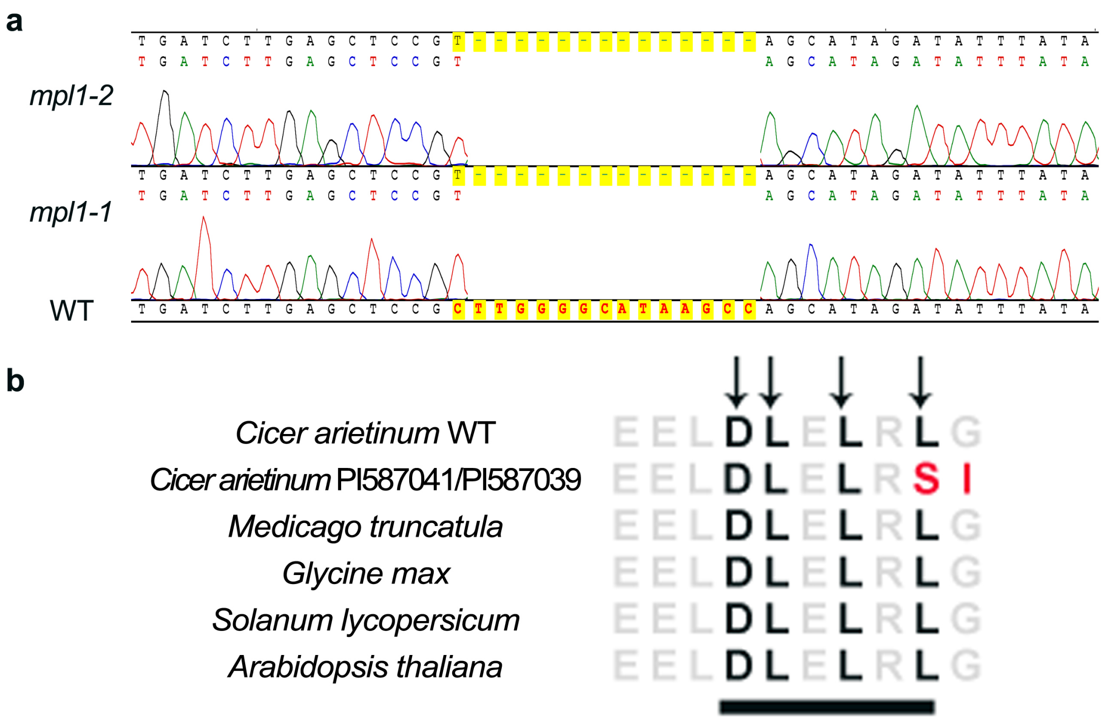

Supplementary Fig. 5. Identification of the causative mutation in the *MPL1* gene. a, Sanger sequencing chromatograms showing mutations in the *MPL1* gene of the *mpl1-1* and *mpl1-2* mutants. **b,** Alignment of the C-terminal partial protein sequences containing DLXLXLX-type EAR motifs of MPL1 homologs from different species. The black arrow indicates the conserved amino acid residue in EAR motif, while induced mutated amino acid residues in *mpl1-1* and *mpl1-2* are marker in bold red.

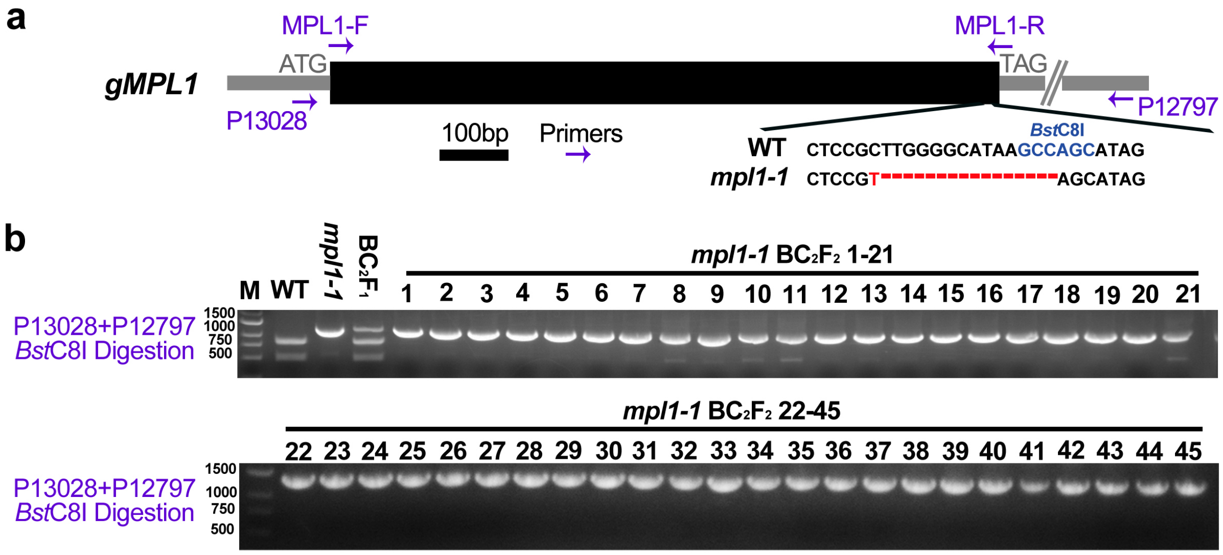

Supplementary Fig. 6. Cosegregation analysis in the F2 population from a cross between the *mpl1-1* mutant and WT. a, Genomic structure of *MPL1*. The black and gray boxes represent exons and the flanking genomic sequences, respectively. Arrows indicate primers used for genotyping and cosegregation analysis. The sequences marked in blue denote *Bst*C8I enzyme digestion recognition site. **b,** Cosegregation analysis of an F2 population derived from *mpl1-1*(♀)×WT (cv. ICCV96029) (♂). 45 out of 183 F2 individuals showing the *mpl1-1* mutant phenotype were homozygous for the mutation. PCR products were amplified from P13028 and P12797 and then digested by the *Bst*C8I enzyme for 2 hours before DNA agarose gel electrophoresis. Similar results were obtained from three independent experiments. Source data are provided as a Source Data file.

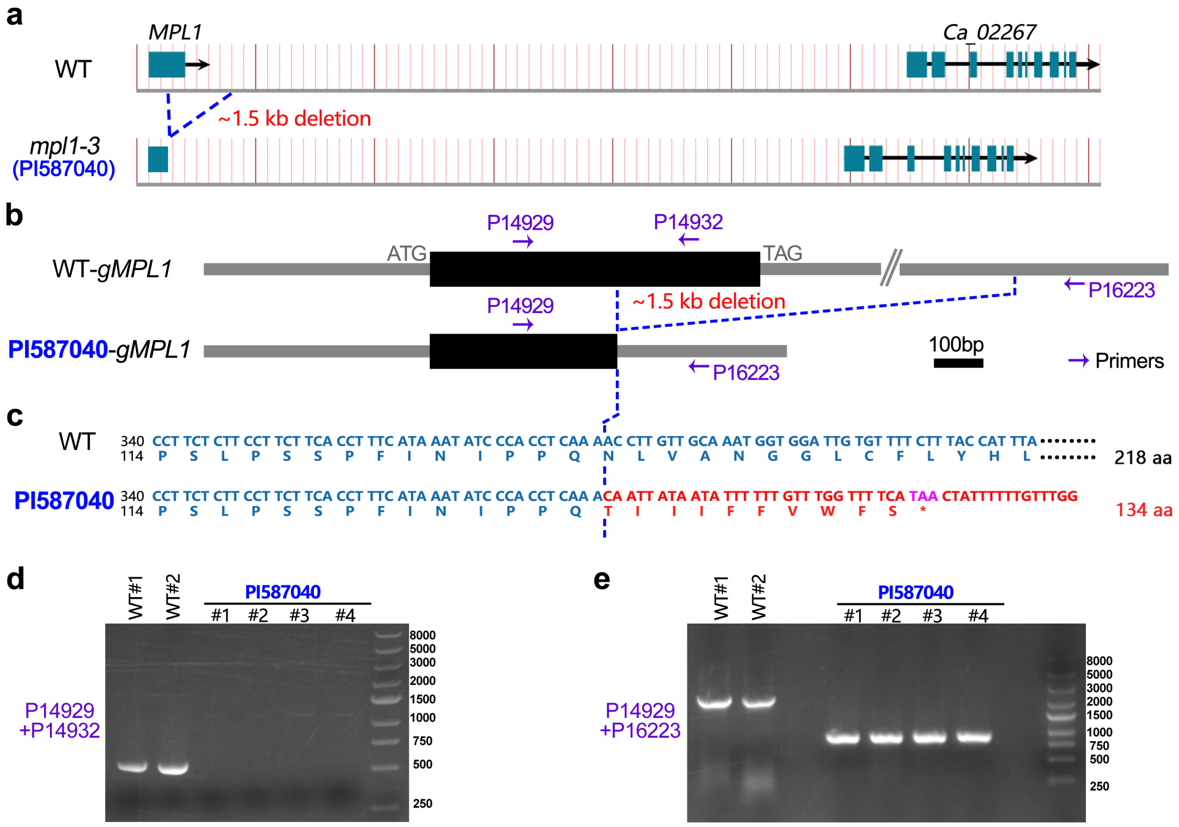

Supplementary Fig. 7. Genotyping analysis of *mpl1-3* (PI587040). a, Chromosomal organization and gene structure of the *MPL1* gene and its neighbor gene *Ca_02267* with exons shown as dark cyan boxes. Arrows indicate the direction of transcription of each gene. The *mpl1-3* carried a 1.5 kb deletion encompassing a portion of the *MPL1* gene. **b,** Genomic structure of *MPL1* in WT and *mpl1-3* (PI587040). The black and gray boxes represent exons and the flanking genomic sequences, respectively. Arrows indicate primers used for genotyping analysis. **c,** A 1.5 kb deletion encompassing a portion of the *MPL1* gene in *mpl1-3* (PI587040) created a premature stop codon. **d,e,** Genotyping analysis of WT and *mpl1-3* (PI587040). Similar results were obtained from three independent experiments. Source data are provided as a Source Data file. The primers are shown in Supplementary Table 3.

a

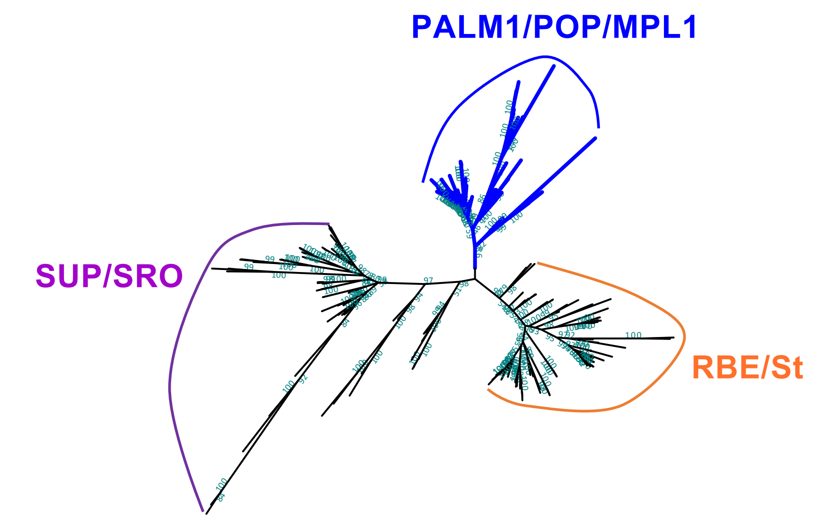

b

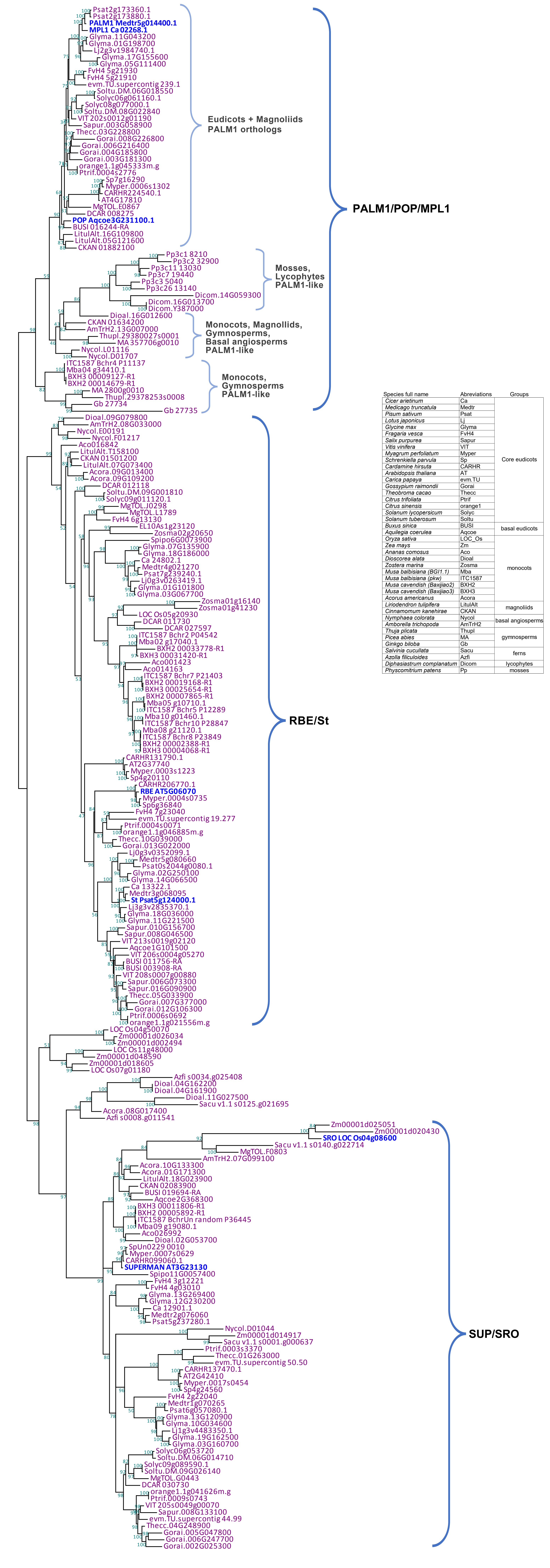

Species full name	Abreviations	Groups
Cicer arietinum	Ca	Core eudicots
Medicago truncatula	Medtr	
Pisum sativum	Psat	
Lotus japonicus	Lj	
Glycine max	Glyma	
Fragaria vesca	FvH4	
Salix purpurea	Sapur	
Vitis vinifera	VIT	
Myagrum perfoliatum	Myper	
Schrenkiella parvula	Sp	
Cardamine hirsuta	CARHR	
Arabidopsis thaliana	AT	
Carica papaya	evm.TU	
Gossypium raimondii	Gorai	
Theobroma cacao	Thecc	
Citrus trifoliata	Ptrif	
Citrus sinensis	orange1	
Solanum lycopersicum	Solyc	
Solanum tuberosum	Soltu	
Buxus sinica	BUSI	basal eudicots
Aquilegia coerulea	Aqcoe	
Oryza sativa	LOC_Os	monocots
Zea mays	Zm	
Ananas comosus	Aco	
Dioscorea alata	Dioal	
Zostera marina	Zosma	
Musa balbisiana (BGI1.1)	Mba	
Musa balbisiana (pkw)	ITC1587	
Musa cavendish (Baxijiao2)	BXH2	
Musa cavendish (Baxijiao3)	BXH3	
Acorus americanus	Acora	
Liriodendron tulipifera	LitulAlt	magnoliids
Cinnamomum kanehirae	CKAN	
Nymphaea colorata	Nycol	basal angiosperms
Amborella trichopoda	AmTrH2	
Thuja plicata	Thupl	gymnosperms
Picea abies	MA	
Ginkgo biloba	Gb	
Salvinia cucullata	Sacu	ferns
Azolla filiculoides	Azfi	
Diphasiastrum complanatum	Dicom	lycophytes
Physcomitrium patens	Pp	mosses

Supplementary Fig. 8. Phylogeny of MPL1 and its homologs from other species constructed using the maximum-likelihood method and bootstrap test with 2000 replicates.

a, A radiation tree of MPL1 and its homologs exhibited 3 different clades. MPL1, PALM1, and POP were grouped together forming a distinct clade (blue) sister to two other clades which include the *Arabidopsis* SUPERMAN (SUP) the RABBIT EARS (RBE) respectively. **b,** The fully annotated tree.

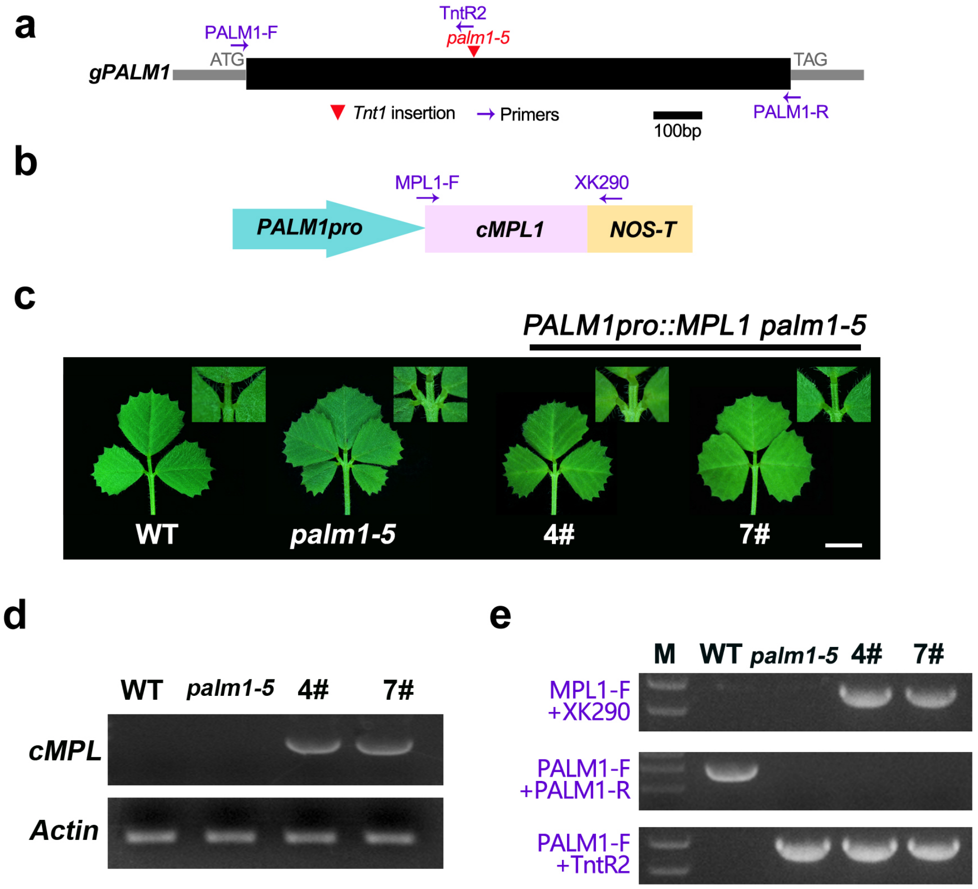

Supplementary Fig. 9. The rescued phenotype of *palm1* mutant by *PALM1pro::MPL1* in *M. truncatula*. a, Genomic structure of *PALM1*. The black and gray boxes represent exons and the flanking genomic sequences, respectively. The red triangle denote *Tnt1* insertion site in *PALM1* of *palm1-5* mutant. Arrows indicate primers used for genotyping. **b,** Schematic diagram of *PALM1pro:cMPL1-NOS-T* construct used for genetic complementary assay. The blue, pink and orange boxes represent the promoter of *PALM*, the coding region of *MPL1* and the NOS terminator, respectively. **c,** Leaf phenotypes of WT, *palm1-5* and two independent rescued lines. Images in each top right corner show close-up views of the adaxial side of the leaflet basal region. Scale bar, 1 cm. **d,** RT-PCR analysis of *MPL1* expression in shoot buds of WT, *palm1-5* and two independent transgenic rescued lines. **e,** Genotyping of the independent transgenic rescued lines. Similar results were obtained from three independent experiments. Source data are provided as a Source Data file. The primers are shown in Supplementary Table 3.

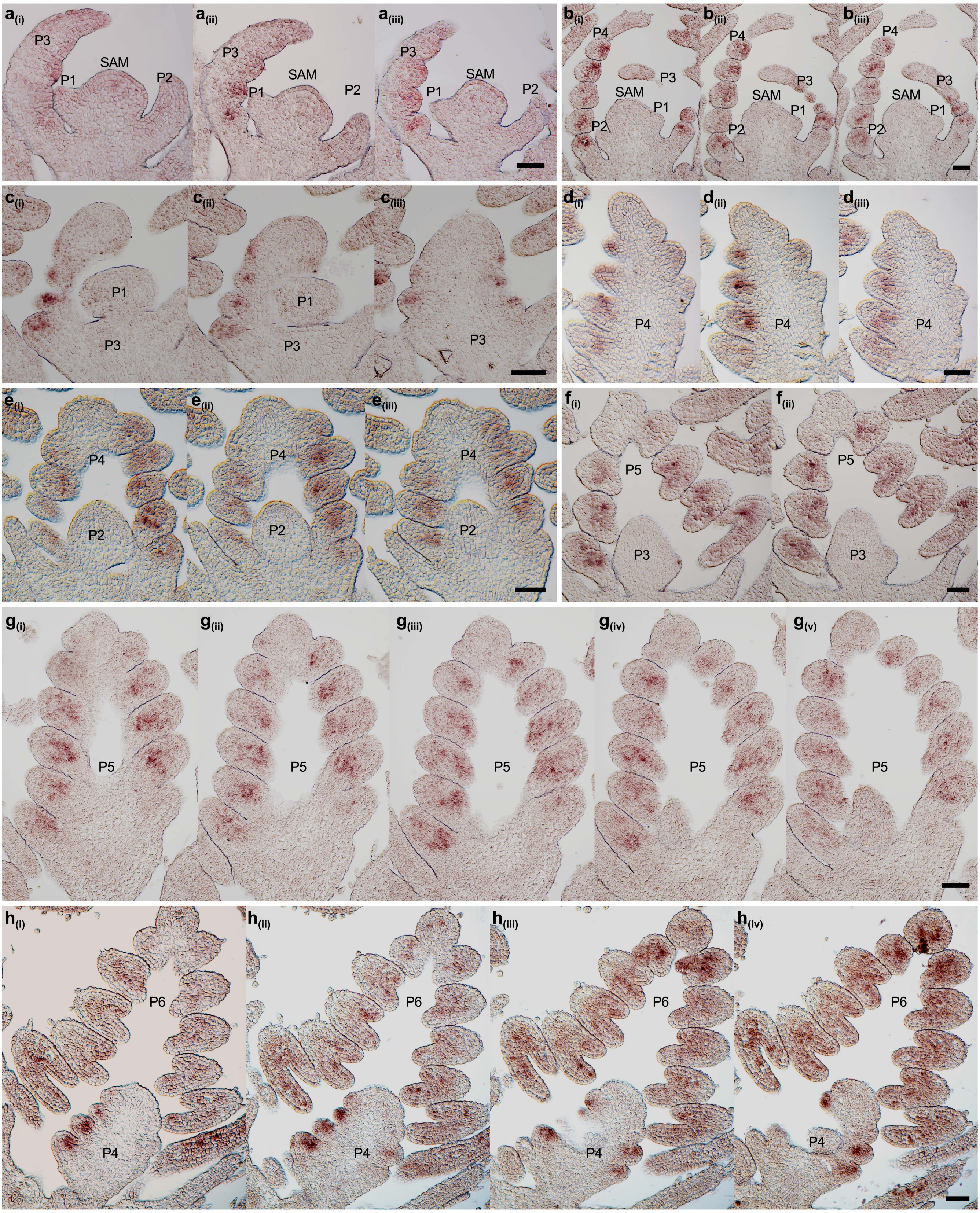

Supplementary Fig. 10. The *MPL1* expression pattern during leaf development detected by RNA *in situ* hybridization. a,b, Longitudinal successive sections of WT vegetative shoot apex. **c-h,** Longitudinal successive sections of leaf primordium at the indicated developmental stages. Similar results were obtained from three independent experiments. Scale bars, 50 μm

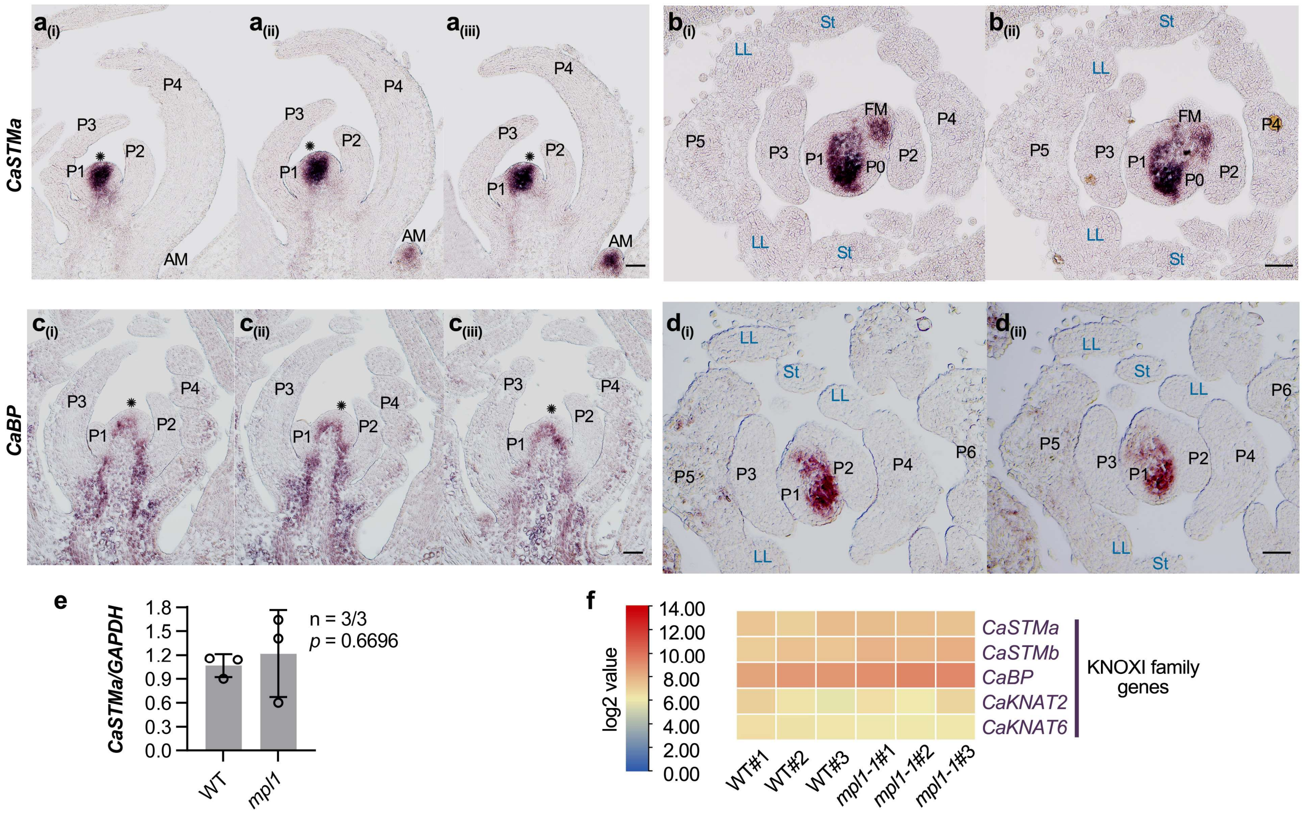

Supplementary Fig. 11. The expression patterns of *CaSTMa* and *CaBP1*, and differential expression analysis of *CaKNOXIs* between WT and the *mpl1-1* mutant. a,b, The expression pattern of *CaSTMa* in successive longitudinal (**a**) and transverse (**b**) sections of SAM with P0~P5 leaf primordia detected by RNA *in situ* hybridization. **c,d,** The expression pattern of *CaBP1* in successive longitudinal (**c**) and transverse (**d**) sections of SAM with P0~P5 leaf primordia. Similar results were obtained from three independent experiments. Scale bars, 50 μm. **e,** RT–qPCR analysis of *CaSTMa* mRNA expression levels in vegetative-shoots between WT and *mpl1-1*. Data shows mean ± SD of 3 biological replicates. The values above bars represent the *p*-value estimated by the two-sided unpaired Student's *t* test for the comparison between WT and the *mpl1-1* mutant. **f,** Differential expression of *CaKNOXIs* in vegetative shoots between WT and *mpl1*, based on RNA-seq transcriptome analysis. A heat map was generated using fragments per kilobase of exon model per million mapped reads (FPKM). WT and *mpl1* biological replicates were plotted in the left and right three columns, respectively.

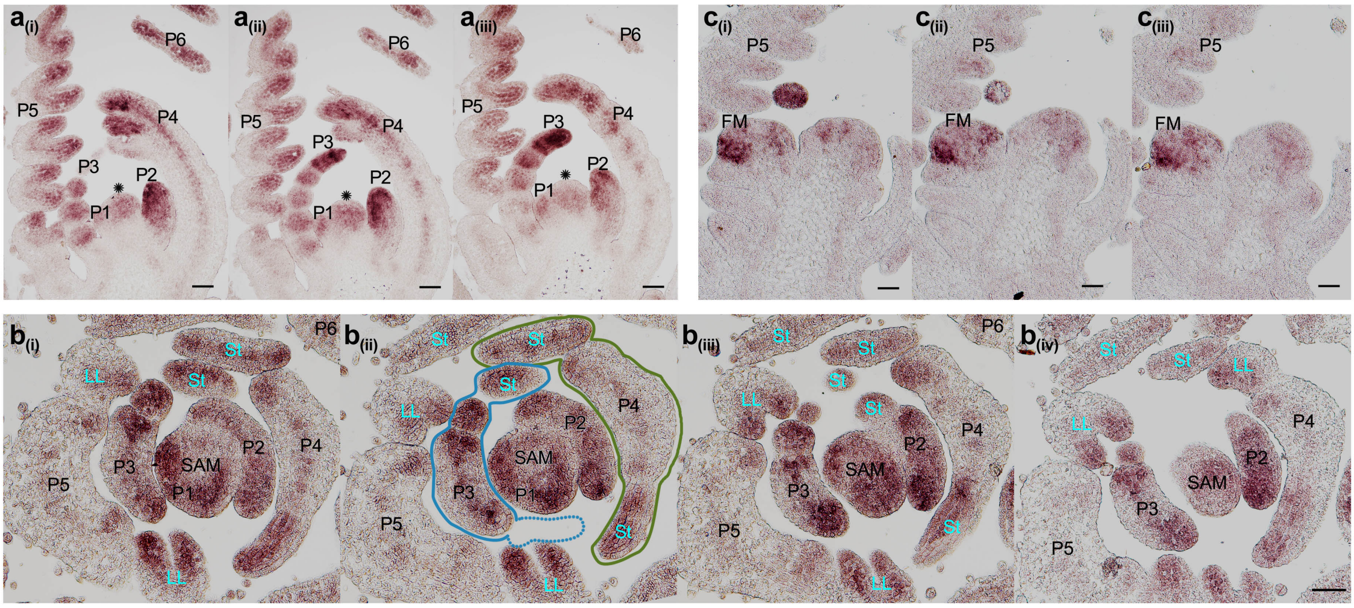

Supplementary Fig. 12. RNA *in situ* hybridization of *CaLFY* in shoot apex of WT chickpea. a, The expression pattern of *CaLFY* in longitudinal successive sections of WT vegetative shoot apex. **b,** The expression pattern of *CaLFY* in transverse successive sections of WT vegetative shoot apex. **c,** The expression pattern of *CaLFY* in longitudinal successive sections of WT reproductive shoot apex. Similar results were obtained from three independent experiments. Scale bars, 50 μm.

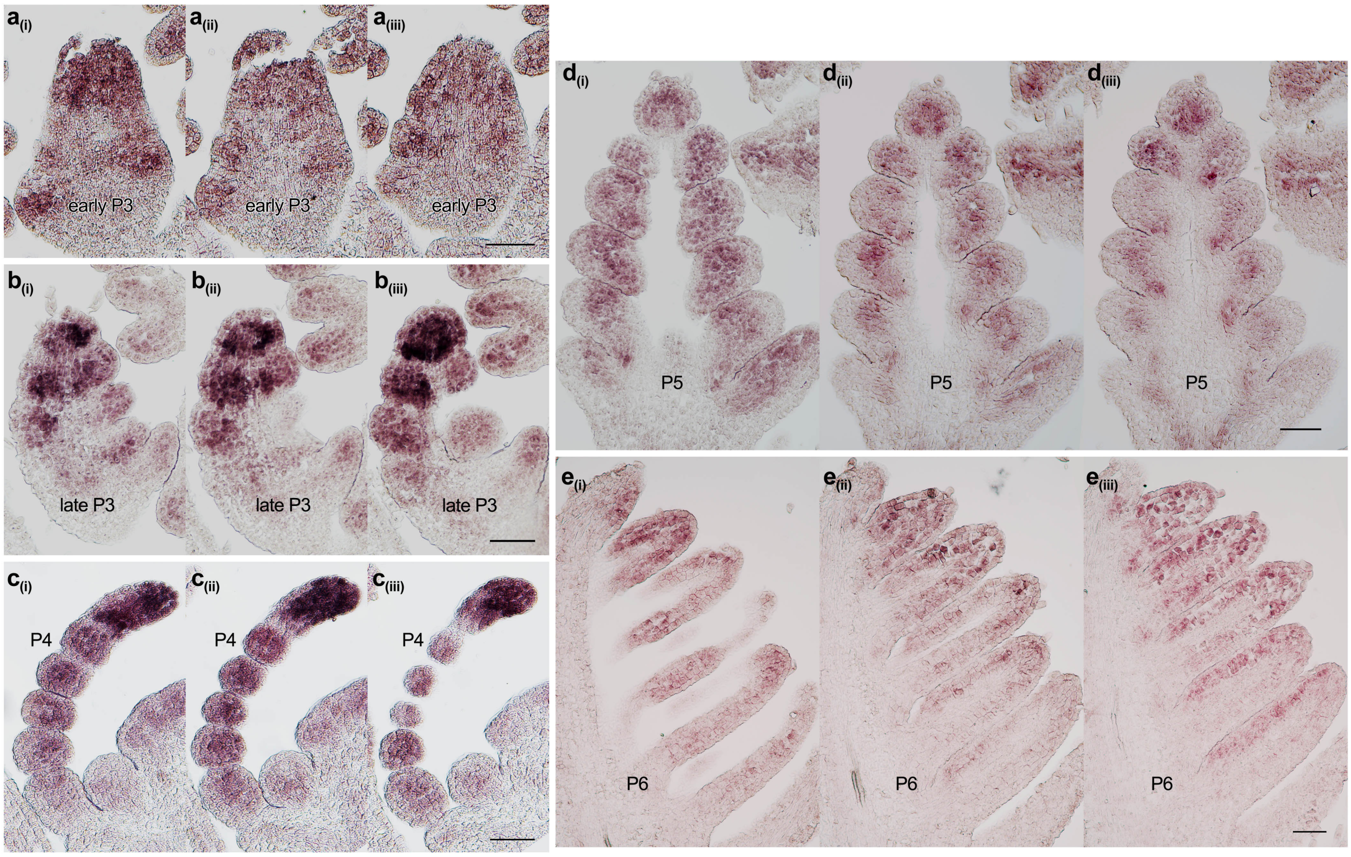

Supplementary Fig. 13. RNA *in situ* hybridization of *CaLFY* during leaf development in WT chickpea. Shown are serial coronal sections through leaf primordia at the early P3 (**a**), late P3 (**b**), and P5 (**d**) stages, and serial sagittal sections through leaf primordia at the P4 (**c**) and P6 (**e**) stages**.** Similar results were obtained from three independent experiments. Scale bars, 50 µm.

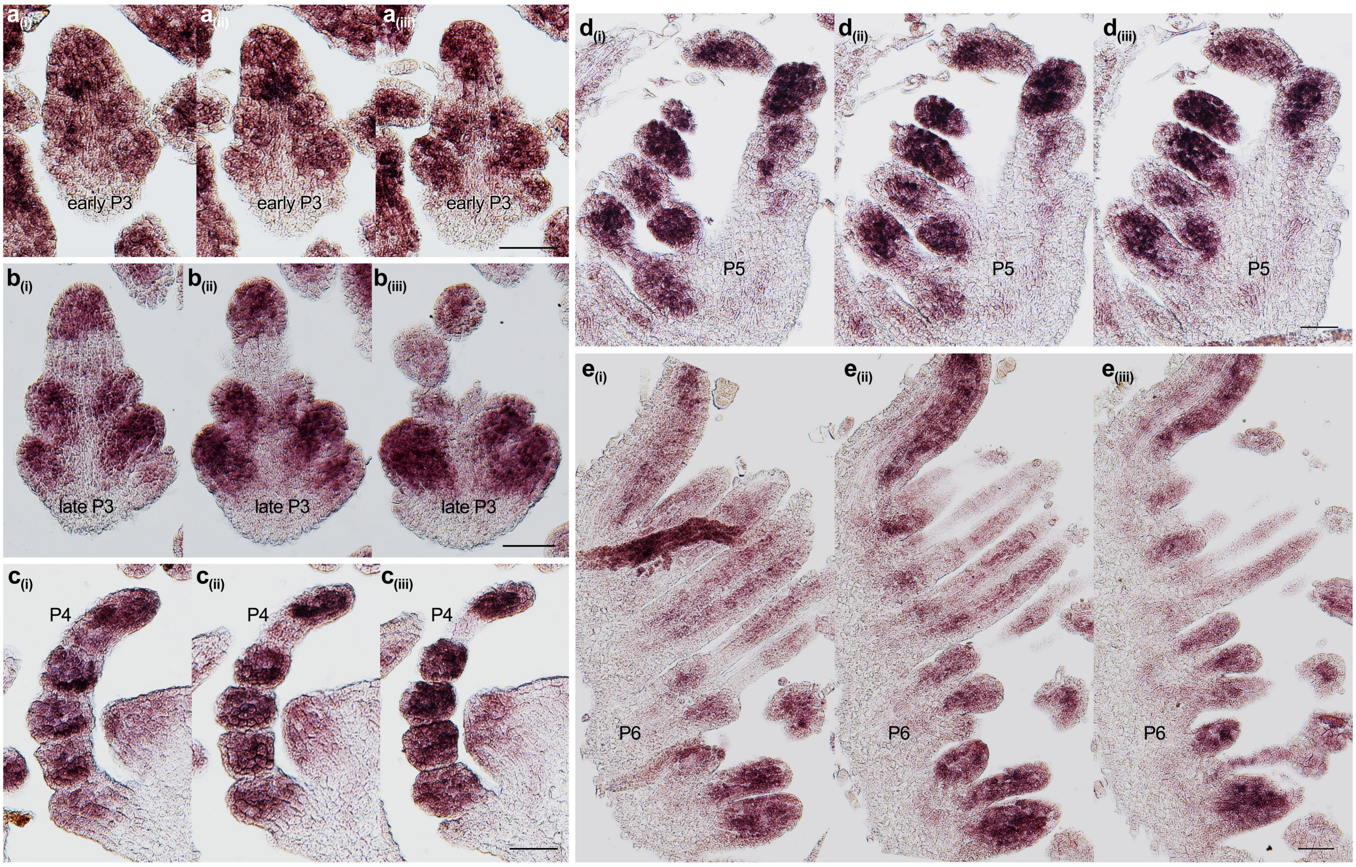

Supplementary Fig. 14. RNA *in situ* hybridization of *CaLFY* during leaf development in the *mpl1-1* mutant of chickpea. Shown are serial coronal sections through leaf primordia at the early P3 (**a**), late P3 (**b**), and P5 (**d**) stages, and serial sagittal sections through leaf primordia at the P4 (**c**) and P6 (**e**) stages**.** Similar results were obtained from three independent experiments. Scale bars, 50 μm.

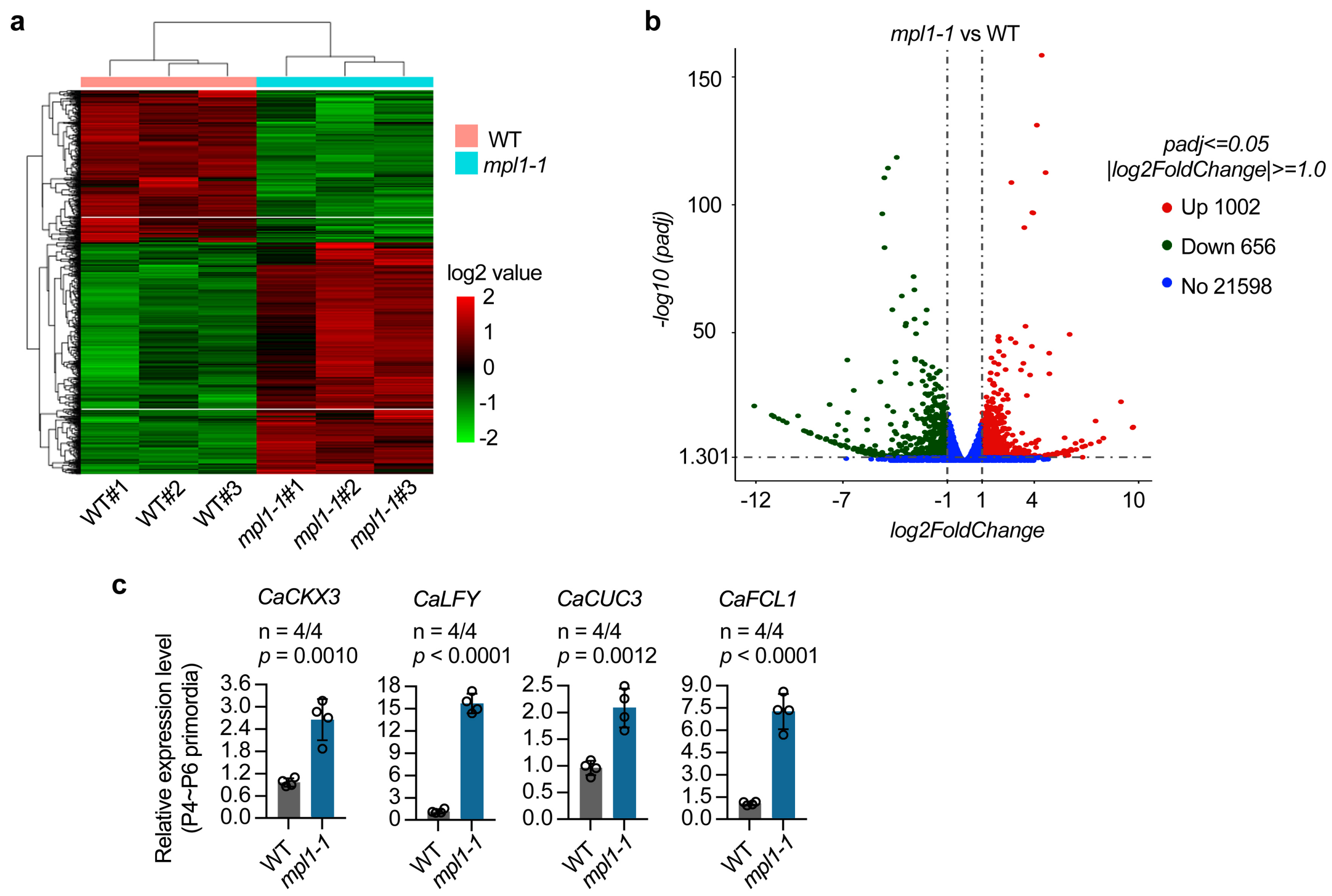

Supplementary Fig. 15. The differentially expressed genes (DEGs) analysis between WT and *mpl1-1*. **a,** The heatmap of normalized FPKM value of all the differentially expressed genes (DEGs) in WT and *mpl1-1* vegetative shoots with three biological replicates. **b,** Volcano plots showing the DEG number in *mpl1-1* compared with WT in two-week-old vegetative shoots (SAM to P6). **c,** RT–qPCR validation of expression differences of certain selected genes between WT and *mpl1-1*. The samples used for the analysis consist of a mixture of leaf primordia at stages P4, P5 and P6 that are dissected from the shoot apex. Data shows mean ± SD of 4 biological replicates. The values above bars represent the *p*-value estimated by the two-sided unpaired Student's *t* test for the comparison between WT and the *mpl1-1* mutant.

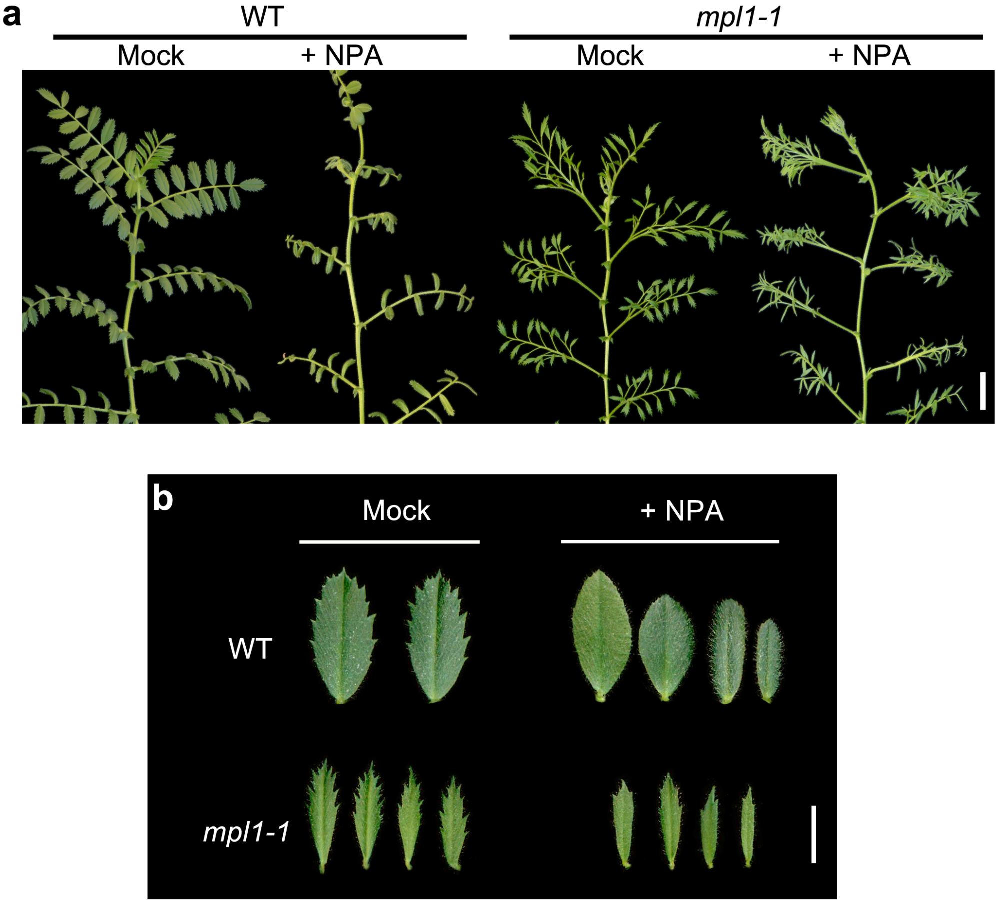

Supplementary Fig. 16. The morphology changes between WT and *mpl1-1* after spraying with H_2O (Mock) and NPA (100 µM). a, Plant growth response to N-1-naphthylphthalamic acid (NPA). Two-week-old plants of WT and *mpl1-1* grown in soil were sprayed by mock or 100 µM NPA once every three day for 15 days, and plants and compound leaves were photographed at 3 weeks after the spraying stopped. Similar results were obtained from three independent experiments. **b,** Leaflets dissected from the 11th node compound leaves of WT and *mpl1-1* after spraying with H_2O (Mock) and NPA (100 µM). Scale bars, 2 cm in **a** and 0.5 cm in **b**.

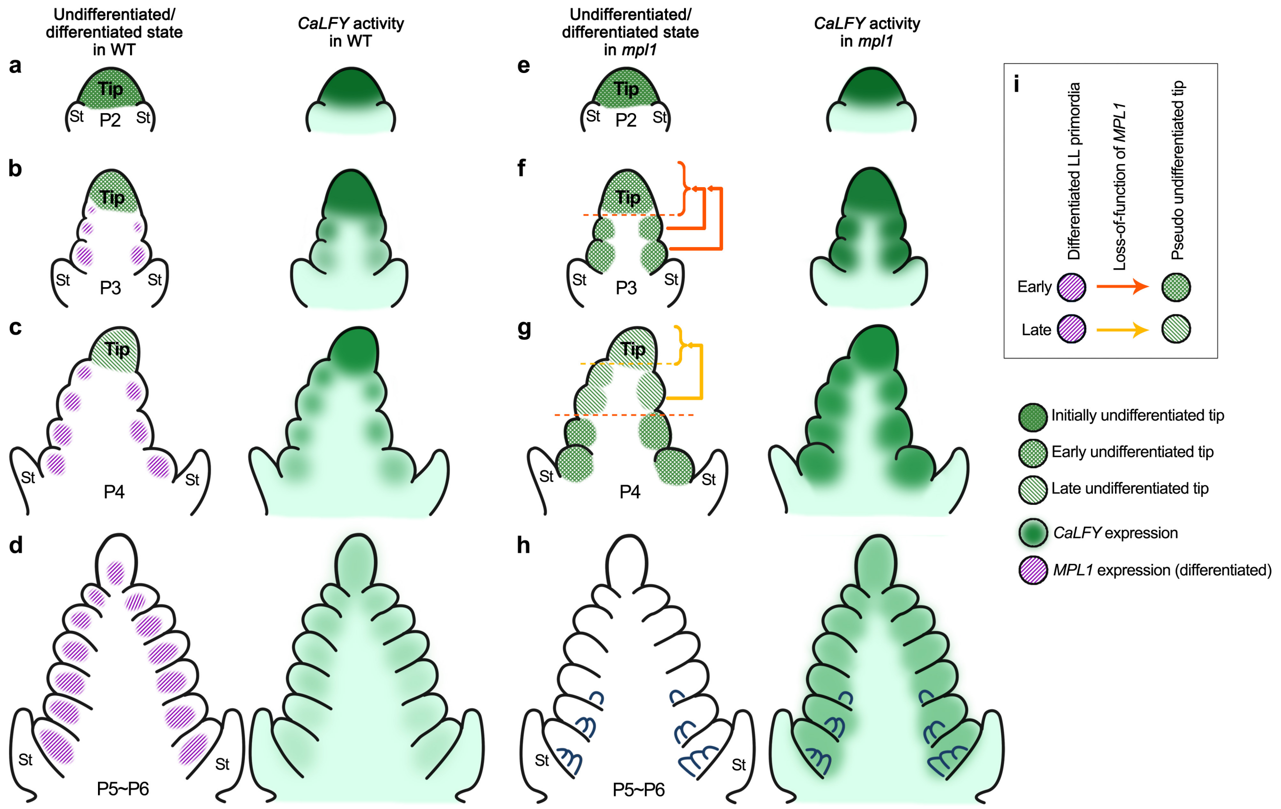

Supplementary Fig. 17. Models for Chickpea leaf development in WT and *mpl1*. a-d, Diagrams of the undifferentiated/differentiated state (left row) and *CaLFY* activity (right row) within compound leaf primordia of WT. **e-h,** Diagrams of the undifferentiated/differentiated state (left row) and *CaLFY* activity (right row) within compound leaf primordia of *mpl1*. **i,** The loss-of-function of *MPL1* converted the LL primordia as "pseudo undifferentiated tips", which have potential to generate new leaflet primordia.

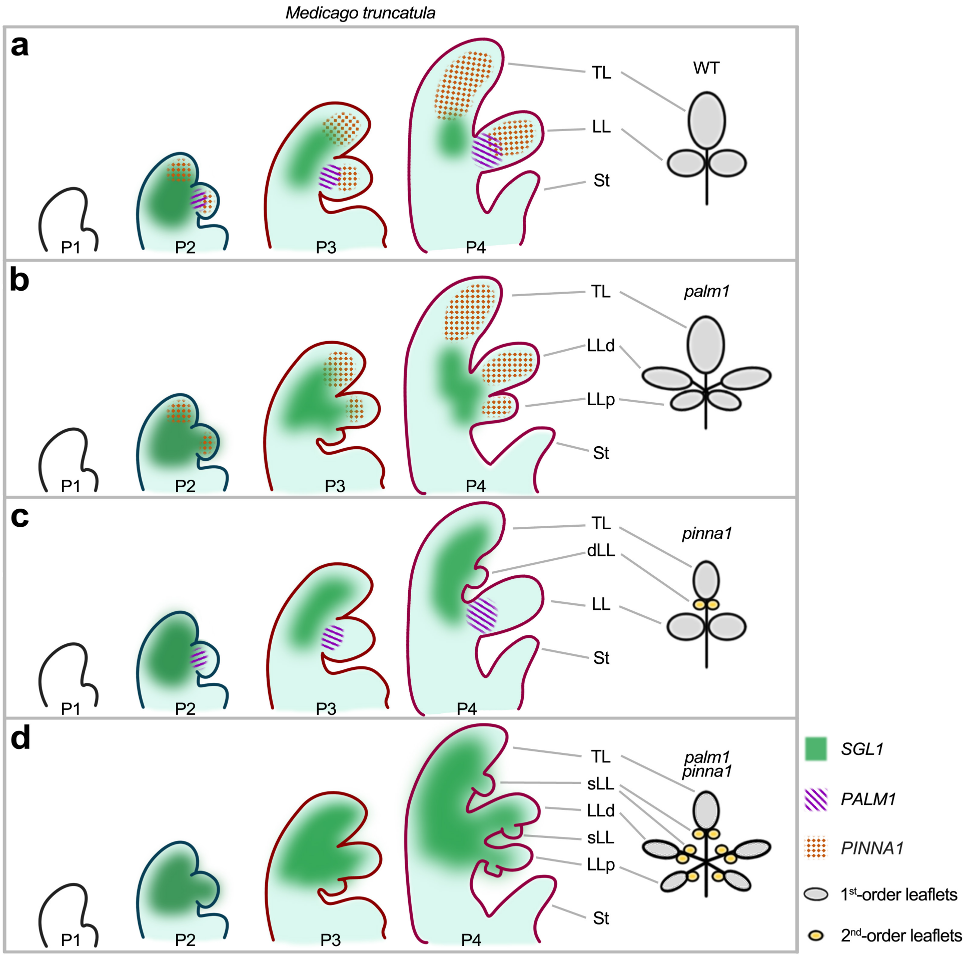

Supplementary Fig. 18. Working Models for leaf development in different mutants of *Medicago truncatula*. **a,** In WT, *PINNA1* and *PALM1* are expressed in different regions to repress *SGL1* expression, maintaining the trifoliate leaf pattern. **b,** In the *palm1* mutant, *SGL1* expression expands to basal regions of the lateral leaflet primordia at the P3 stage, inducing two extra leaflet primordia emerging from there. This converts the trifoliate pattern into a palmate pattern. **c,** In the *pinna1* mutant, the *SGL1* expression was upregulated and expanded in the terminal leaflet primordia at the P4 stage, inducing ectopic leaflets in the terminal leaflet region. This changes the trifoliate pattern into a pinnate pattern. **d,** In the *palm1 pinna1* double mutant, the *palm1* mutation is responsible for the palmate pattern of five 1st-order leaflets, while the *pinna1* mutation resulted the upregulation and expansion of *SGL1* expression in all 1st-order leaflet primordia, which promotes the formation of all 2nd-order leaflets, leading to high-order compound leaves.
