# Supplementary material for: Control of compound leaf patterning by MULTI-PINNATE LEAF1 (MPL1) in chickpea
Source: Nat Commun. 2023 Dec 7;14:8088. doi: 10.1038/s41467-023-43975-9 (PMC10703836; doi:10.1038/s41467-023-43975-9)
Supplement: Supplementary file 3 — Description of Additional Supplementary Files [file 41467_2023_43975_MOESM3_ESM.pdf]

Description of Additional Supplementary Files

File Name: Supplementary Data 1.
Description: The information of 5 candidate genes on chromosome 8 by BSA-analysis.

File Name: Supplementary Data 2.
Description: RNA-seq read statistics of WT and the *mpl1-1* mutant.

File Name: Supplementary Data 3.
Description: Selected DEGs in RNA-Seq analysis of the *mpl1-1* mutant and WT.

File Name: Supplementary Data 4.
Description: 656 significantly down-regulated genes in *mpl1-1* mutant compared to WT.

File Name: Supplementary Data 5.
Description: 1002 significantly up-regulated genes in *mpl1-1* mutant compared to WT.

File Name: Supplementary Data 6.
Description: PCR primer sequences used in this study.

File Name: Supplementary Data 7.
Description: Alignment of all identified MPL1 homologs from different species (To open this file, you need to rename it as a .aln file and then open it by ClustalX 2.1).

File Name: Supplementary Data 8.
Description: Data of a full Maximum likelihood tree of MPL1 homologs (To open this file, you need to rename it as a .nwk file, and then open it by MEGA7).
